# Supplementary material for: Role of Seipin in Human Diseases and Experimental Animal Models
Source: Biomolecules. 2022 Jun 17;12(6):840. doi: 10.3390/biom12060840 (PMC9221541; doi:10.3390/biom12060840)
Supplement: Supplementary file 1 [file biomolecules-12-00840-s001.zip › biomolecules-1741998-supplementary.pdf]

biomolecules

MDPI

Table S1. Case reports (n < 9) of diseases caused by *BSCL2* gene mutations.

Studies	Phenotype	OMIM/Inheritance	Patients	Mutation classification	Clinical features and end-organ complications
Rajab et al. 2002[1]	CGL2	269700/AR	4 patients	NA	Metabolic disorders (diabetes mellitus, hyperlipidaemia, hypercholesterolaemia)<br>Changes in physical appearances (acanthosis nigricans, hirsutism, muscle hypertrophy, enlarged hands and feet, abundant "kinky'' scalp hair, premature greying of the hair, odd shaped skull, and ''coarse'' facial features)<br>Organ injury induced by metabolic disorders (hepatomegaly, splenomegaly, and nephropathy)<br>Changes in reproductive system (precocious puberty)<br>Others (significant developmental delay, accelerated growth, and advanced bone age in the first year of life)
Bhayana et al. 2002[2]	CGL2	269700/AR	1 patient	Nonsense	Metabolic disorders (diabetes mellitus, hyperlipidaemia, hypercholesterolaemia)<br>Changes in physical appearances (hirsutism, muscle hypertrophy, enlarged hands and feet, coarse facial features, hollowed cheeks, triangular and progeroid appearance)<br>Organ injury induced by metabolic disorders (myocardial hypertrophy, hepatomegaly, splenomegaly, umbilical hernia)<br>Changes in reproductive system (clitoromegaly and Tanner stage 2 breast development.)<br>Neurodegenerative phenotypes (mild to moderate intellectual impairment, waddling gait)
Ebihara et al. 2004[3]	CGL2	269700/AR	3 patients	Nonsense	Metabolic disorders (diabetes mellitus, insulin resistance, hypoleptinaemia)<br>Changes in physical appearances (acanthosis nigricans)<br>Organ injury induced by metabolic disorders (fatty liver)<br>Changes in reproductive system (oligomenorrhea and polycystic ovary)<br>Neurodegenerative phenotypes (mental retardation)
Raygada et al. 2005[4]	CGL2	269700/AR	2 patients	NA	Metabolic disorders (diabetes mellitus, insulin resistance, hypertriglyceridaemia)<br>Changes in physical appearances (hirsutism, acromegaloid features, protruding abdomen, hollow cheeks, prominent ears, and possible rachitic changes)<br>Organ injury induced by metabolic disorders (hepatomegaly, fatty liver, liver cirrhosis, hypertrophic cardiomyopathy, and enlarged kidneys)<br>Changes in reproductive system (precocious puberty)

					Neurodegenerative phenotypes (intellectual impairment, impaired motor skills)<br>Others (chronic nasal congestion, enlarged tonsils and adenoids)
Mandal et al. 2006[5]	CGL2	269700/AR	3 patients	Nonsense or frameshift	Metabolic disorders (insulin resistance, hypertriglyceridaemia)<br>Changes in physical appearances (acanthosis nigricans, hirsutism, muscle hypertrophy, abdominal distension, abnormal facies, and large superficial veins)<br>Organ injury induced by metabolic disorders (hepatosplenomegaly, absence of one kidney)<br>Changes in reproductive system (hypertrophic genitalia)<br>Neurodegenerative phenotypes (mental retardation)<br>Others (they are found to be prone to upper respiratory tract infections and skin abscesses, but respond well to oral antibiotics)
Jin et al. 2007[6]	CGL2	269700/AR	1 patient	Nonsense	Metabolic disorders (diabetes mellitus, insulin resistance, hypertriglyceridaemia)<br>Changes in physical appearances (acanthosis nigricans, hirsutism, muscle hypertrophy, enlarged hands and feet)<br>Organ injury induced by metabolic disorders (hepatosplenomegaly, hepatocirrhosis, a left ventricle hypertrophy, and a cardiac murmur)<br>Changes in reproductive system (crassitude of the penis)<br>Neurodegenerative phenotypes (mild mental retardation)<br>Others (accelerated growth, voracious appetite, and gum bleeding repeatedly)
Shirwalkar et al. 2008[7]	CGL2	269700/AR	1 patient	Frameshift	Metabolic disorders (diabetes mellitus, insulin resistance, hypertriglyceridaemia)<br>Changes in physical appearances (acanthosis nigricans, hirsutism, muscle hypertrophy, and peculiar pinched facies)<br>Organ injury induced by metabolic disorders (hepatosplenomegaly, mild cardiomegaly, and renal failure)<br>Changes in reproductive system (polycystic ovary syndrome)<br>Others (advanced bone age)
Friguls et al. 2009[8]	CGL2	269700/AR	1 patient	Nonsense	Metabolic disorders (hypoleptinaemia, hypertension)<br>Changes in physical appearances (hirsutism, prominent muscles and veins, large hands and feet, and abdominal distension)<br>Organ injury induced by metabolic disorders (hepatomegaly, severe liver steatosis, hypertrophic cardiomyopathy, and heart failure)<br>Neurodegenerative phenotypes (mild mental retardation)<br>Others (pancreatitis)

Miranda et al. 2009[9]	CGL2	269700/AR	7 patients	Nonsense, missense, frameshift, or unknown	Metabolic disorders (hypertriglyceridaemia, insulin resistance, hypoleptinaemia)<br>Changes in physical appearances (acanthosis nigricans)<br>Organ injury induced by metabolic disorders (hepatomegaly, cardiomyopathy)
Wu et al. 2009[10]	CGL2	NA/AD	1 patient	Nonsense and frameshift	Metabolic disorders (diabetes mellitus, hypertriglyceridaemia)<br>Changes in physical appearances (acanthosis nigricans, hirsutism, muscular hypertrophy, acromegaloid features, and hollow cheeks,)<br>Neurodegenerative phenotypes (generalised dystonia)
Roth et al. 2010[11]	CGL2	269700/AR	1 patient	NA	Metabolic disorders (diabetes mellitus, hypertriglyceridaemia, hypertension)<br>Changes in physical appearances (acanthosis nigricans, progeric in appearance, prominent calf muscles, enophthalmus, prominent forehead, eyebrows and mandible, big ears, several teeth missing, caries in many teeth. Hair on the side of the face and on the chin, umbilical hernia, high arched soles, contracture of joints)<br>Organ injury induced by metabolic disorders (hepatosplenomegaly, III/VI holosystolic murmur)<br>Neurodegenerative phenotypes (brisk patellar tendon reflexes)<br>Changes in reproductive system (Tanner II stage breasts, Tanner V stage pubic hair, clitoris prominent)
Huang et al. 2010[12]	CGL2	269700/AR	1 patient	Frameshift	Metabolic disorders (insulin resistance, hypertriglyceridaemia)<br>Changes in physical appearances (prominent musculature, generalised eruptive xanthomas)<br>Organ injury induced by metabolic disorders (hepatomegaly, severe liver steatosis, and periportal necrosis)
Jeninga et al. 2012[13]	CGL2	269700/AR	1 patient	NA	Metabolic disorders (diabetes mellitus, hypertriglyceridaemia, and low high-density lipoprotein cholesterol)<br>Changes in physical appearances (acanthosis nigricans, muscle hypertrophy, large hands and feet)<br>Organ injury induced by metabolic disorders (enlarged liver, liver steatosis, and hypertrophic cardiomyopathy)<br>Changes in reproductive system (enlarged external genitalia)<br>Neurodegenerative phenotypes (mild mental retardation)<br>Others (upper respiratory tract infection, elevated urinary organic acid)
Rahman et al. 2013[14]	CGL2	269700/AR	2 patients	Frameshift	Changes in physical appearances (acanthosis nigricans, muscular hypertrophy, prominent veins, rough dry skin and umbilical protrusion)

					Organ injury induced by metabolic disorders (moderate hepatomegaly with mild splenomegaly, hypertrophic cardiomyopathy)<br>Changes in reproductive system (moderate genital enlargement)<br>Others (hyper-density of partial bones)
Jiang et al. 2014[15]	CGL2	269700/AR	1 patient	Nonsense	Metabolic disorders (diabetes mellitus)<br>Changes in physical appearances (acanthosis nigricans, extremely muscular and acromegalic appearance, umbilical hernia)<br>Changes in reproductive system (teratozoospermia, enlarged penis)
Schuster et al. 2014[16]	CGL2	269700/AR	3 patients	Not available	Metabolic disorders (diabetes mellitus)<br>Changes in physical appearances (acanthosis nigricans, muscular hypertrophy, acromegaloid appearance, large hands and feet)<br>Organ injury induced by metabolic disorders (enlarged liver and spleen)<br>Neurodegenerative phenotypes (mental retardation, spastic gait)<br>Others (skeletal abnormalities)
Haghighi et al. 2016[17]	CGL2	269700/AR	5 patients	Nonsense, frameshift, or unknown	Metabolic disorders (diabetes mellitus, insulin resistance, hypertriglyceridaemia, hypertension)<br>Changes in physical appearances (acanthosis nigricans, hypertrichosis, acromegaloid features, muscle hypertrophy, hernia, large ears, triangular facies)<br>Organ injury induced by metabolic disorders (hepatomegaly, elevated liver enzymes, steatohepatitis, splenomegaly, cardiomyopathy, nephropathy)<br>Changes in reproductive system (changes in genitalia, retractile testes, breast enlargement, clitoromegaly, intrauterine growth restriction)<br>Neurodegenerative phenotypes (intellectual disability)<br>Others (bone cysts)
Bhujel et al. 2016[18]	CGL2	269700/AR	1 patient	NA	Metabolic disorders (diabetes mellitus)<br>Changes in physical appearances (acanthosis nigricans, hypertrichosis)<br>Organ injury induced by metabolic disorders (fatty liver, hypertrophic cardiomyopathy)<br>Changes in reproductive system (precocious puberty)<br>Dental changes (severe crowding of maxillary and mandibular arches, labial exclusion of 33 and 43, aberrant crown morphology, Caries, generalised plaque induced gingivitis)<br>Others (growth disorder, constipation, and thrombocytopenia)
Opri et al. 2016[19]	CGL2	269700/AR	3 patients	Frameshift	Metabolic disorders (hypertriglyceridaemia)<br>Changes in physical appearances (acanthosis nigricans, muscle hypertrophy)

					Organ injury induced by metabolic disorders (steatohepatitis, hypertrophic cardiomyopathy)<br>Neurodegenerative phenotypes (progressive myoclonus epilepsy, delayed language ability, intention myoclonus, pyramidal signs, and loss of language, a profound intellectual impairment and dystonic tetraplegia with continuous myoclonus)
Teboul-Coré et al. 2016[20]	CGL2	269700/AR	2 patients	Nonsense	diffuse osteosclerosis, well-defined osteolytic lesions sparing the axial skeleton
Su et al. 2017[21]	CGL2	269700/AR	3 patients	Nonsense, frameshift, or unknown	Metabolic disorders (diabetes mellitus, insulin resistance, severe hypertriglyceridaemia)<br>Changes in physical appearances (acanthosis nigricans, hirsutism, and muscular hypertrophy)<br>Organ injury induced by metabolic disorders (hepatomegaly, fatty liver, and cardiomyopathy)<br>Neurodegenerative phenotypes (mild intellectual impairment with developmental language disorders, emotional excitability and hyperactivity)
Chen et al. 2017[22]	CGL2	269700/AR	2 patients	Missense, or frameshift	Metabolic disorders (hypertriglyceridaemia, hypercholesterolaemia, and low high-density lipoprotein cholesterol concentration)<br>Changes in physical appearances (acanthosis nigricans, hirsutism, muscular hypertrophy, enlarged hands and feet, and empty cheeks)<br>Organ injury induced by metabolic disorders (hepatomegaly, bilateral renal hypertrophy)<br>Changes in reproductive system (macropenis)
Lima et al. 2017[23]	CGL2	269700/AR	8 patients	NA	high serum sclerostin and good bone microarchitecture
Akinci et al. 2017[24]	CGL2	269700/AR	8 patients	Nonsense or unknown	Peripheral neuropathy, neuropathic diabetic foot ulcer, muscular symptoms, bone cysts
Purizaca-Rosillo et al. 2017[25]	CGL2	269700/AR	5 patients	NA	Metabolic disorders (diabetes mellitus, hypertriglyceridaemia)<br>Changes in physical appearances (acanthosis nigricans, muscular prominence, prominent veins, premature greying of hair, striking aged appearance, and mild anaemia)<br>Organ injury induced by metabolic disorders (abnormal liver enzyme)<br>Neurodegenerative phenotypes (mild intellectual disability)<br>Developmental delay
Gonzalo et al. 2017[26]	CGL2	269700/AR	1 patient	NA	Metabolic disorders (diabetes mellitus, mixed dyslipidaemia)<br>Changes in physical appearances (acanthosis nigricans, hirsutism, typical triangular facies, and stiffness in joints)

					Organ injury induced by metabolic disorders (hepatomegaly, advanced portal fibrosis)<br>Changes in reproductive system (clitorimegaly)<br>Neurodegenerative phenotypes (psychomotor retardation and moderate cognitive impairment)
Ponte et al. 2018[27]	CGL2	269700/AR	5 patients	NA	Metabolic disorders (insulin resistance, hypertriglyceridaemia, and hypoleptinaemia)<br>Others (cardiovascular autonomic neuropathy)
Liu et al. 2019[28]	CGL2	269700/AR	4 patients	Nonsense, frameshift, or unknown	Metabolic disorders (insulin resistance, hypertriglyceridaemia, hypoleptinaemia)<br>Changes in physical appearances (acanthosis nigricans, hypertrichosis, muscular hypertrophy, acromegaloid changes, triangular face, and umbilical hernia)<br>Organ injury induced by metabolic disorders (hepatomegaly, elevated liver enzymes, and splenomegaly)<br>Neurodegenerative phenotypes (mildly delayed mental development, delayed language ability)
Lima et al. 2019[29]	CGL2	269700/AR	1 patient	Frameshift	Metabolic disorders (diabetes mellitus, insulin resistance, hypertriglyceridaemia, hypoleptinaemia)<br>Changes in physical appearances (acanthosis nigricans)<br>Organ injury induced by metabolic disorders (hepatomegaly)<br>Others (papillary thyroid carcinoma)
Zhang et al. 2019[30]	CGL2	269700/AR	1 patient	Frameshift	Progressive myoclonic epilepsy
Serino et al. 2019[31]	CGL2	269700/AR	1 patient	Nonsense	Metabolic disorders (hypertriglyceridaemia, hypertransaminasaemia)<br>Organ injury induced by metabolic disorders (hepatic steatosis)<br>Seizures (generalised tonic seizures and episodes of loss of consciousness, perioral cyanosis, and bilateral eye retropulsion)<br>Neurodegenerative phenotypes (moderate psychomotor delay with speech impairment, severe hyperactivity, and ataxic gait)
Qin et al. 2019[32]	CGL2	269700/AR	1 patient	NA	Metabolic disorders (diabetes mellitus, hypertriglyceridaemia)<br>Changes in physical appearances (acanthosis nigricans)
Yamamoto et al. 2019[33]	CGL2	269700/AR	1 patient	NA	Metabolic disorders (diabetes mellitus, insulin resistance)<br>Changes in physical appearances (acanthosis nigricans, enlarged hands and feet, and prominent mandibular bone)<br>Multiple bone lytic and pseudo-osteopoikilosis lesions limited to the hands and feet
Sánchez-Iglesias et	CGL2	269700/AR	2 patients	Frameshift	Metabolic disorders (hypertriglyceridemia,)<br>Changes in physical appearances (triangular facies)

al. 2019[34]					Neurodegenerative phenotypes (intellectual deficiency, severe language impairment, myoclonic epilepsy, and died in the childhood)
Poisson et al. 2019[35]	CGL2	269700/AR	1 patient	NA	Severe autistic regression in infancy<br>Lethal atypical parkinsonism in adulthood
Ferranti et al. 2020[36]	CGL2	269700/AR	2 patients	NA	Changes in physical appearances (coarse facial features, synophrys, bulbous nasal tip, large ear pinnae, wide mouth, long fingers and toes, and hypertrichosis)<br>Progressive myoclonic epilepsy
Yang et al. 2022[37]	CGL2	269700/AR	1 patient	Nonsense	Metabolic disorders (neonatal onset diabetes, hyperlipidemia)<br>Organ injury induced by metabolic disorders (hepatic steatosis)
Rohkamm et al. 2007[38]	dHMN-V	619112/AD	2 patients	Missense	Weakness and atrophy of the distal muscle groups<br>Foot deformity (pes cavus)<br>Abnormal patellar and achilles tendon reflex<br>Gait (mild unsafeness of gait)<br>Sensory loss (foot ulcerations and infections complicated)<br>Others (shooting and lancinating pain in the lower limbs)
	SS	270685/AD	1 patient	Missense	Weakness and atrophy of the distal muscle groups<br>Foot deformity (pes cavus)<br>Abnormal patellar and achilles tendon reflex<br>Others (shooting pains in both legs and cramps in both feet)
	SS	Sporadic cases	1 patient	Missense	Weakness and atrophy of the distal muscle groups (developed leg spastic paraparesis)<br>Gait (spastic)<br>Brisk patellar reflexes and abnormal tendon reflexes<br>Pyramidal tract damage (increased muscle tone)<br>Sensory deficits
Bienfait et al. 2007[39]	CMT2	NA/AD	3 patients	Missense	Retained or brisk reflexes<br>Extensor plantar response
Cho et al. 2007[40]	Overlapping SS-dHMN	NA/AD	3 patients	Missense	Weakness and atrophy of the distal muscle groups<br>Foot deformity (pes cavus, severe spastic equinovarus deformities)<br>Gait (walking difficulties and frequent falling)<br>Pyramidal tract damage (Babinski signs)<br>Others (ankle deformity)
Cafforio et al. 2008[41]	SS	270685/AD	3 patients	Missense	Weakness and atrophy of the distal muscle groups<br>Foot deformity (pes cavus, hammertoes deformities)<br>Gait (severe paraparetic spastic gait)<br>Pyramidal tract damage (Brisk deep tendon reflexes at lower limbs)<br>Others (severe hearing loss)
Chen et al. 2009[42]	dHMN-II	NA/AD	6 patients	Missense	Predominant weakness of lower extremities<br>Foot deformity (pes cavus)

					Pyramidal tract damage (Babinski signs)
	dHMN-V	619112/AD	3 patients	Missense	Predominant atrophy of hands<br>Foot deformity (pes cavus)<br>Pyramidal tract damage (Patellar tendon reflexes)
Rakocević-Stojanović et al. 2010[43]	dHMN-V	619112/AD	1 patient	Missense	Weakness and atrophy of the distal muscle groups (prominent involvement of legs muscles)<br>Foot deformity (pes cavus)<br>Hyporeflexia in legs<br>Gait (steppage gait, unable to walk on her heels)
	SS	270685/AD	1 patient	Missense	Weakness and atrophy of the distal muscle groups (prominent involvement of hands muscles)<br>Foot deformity (equinovarus feet and ankle deformity)<br>Generalised hyperreflexia with the exception of absent ankle tendon reflexes<br>Gait (mild steppage gait)<br>Pyramidal tract damage (Babinski signs)
	NA	NA	1 patient	Missense	Asymptomatic
Luigetti et al. 2010[44]	CMT2	NA/AD	3 patients	Missense	Weakness and atrophy of the distal muscle groups (prominent involvement of legs muscles)<br>Foot deformity (severe equinovarus deformities, bilateral pes cavus)<br>Generalised hyperreflexia with the exception of absent ankle tendon reflexes<br>Gait (abnormalities)<br>Pyramidal tract damage (Chaddock and Babinski signs, brisk tendon reflexs)<br>Sensory loss
Cen et al. 2015[45]	SS	270685/AD	4 patients	Missense	Weakness and atrophy of the distal muscle groups<br>Hands deformity (claw hands)<br>Gait (spastic)<br>Pyramidal tract damage (bilateral Babinski signs, hypertonia, and hyperreflexia)
Ollivier et al. 2015[46]	Seipinopathy	NA/AD	2 patients	Missense	Weakness and atrophy of the distal muscle groups (rest tremor in the right hand)<br>Foot deformity (pes cavus)<br>Gait (loss of arm swing and amimic)<br>Pyramidal tract damage (bilateral Babinski signs, brisk tendon reflexes in the lower limbs)
Hsiao et al. 2016[47]	CMT2	NA/AD	2 patients	Missense	Weakness and atrophy of the distal muscle groups<br>Gait (Steppage, spastic)<br>Brisk knee jerks
	dHMN	NA/AD	1 patient	Missense	Weakness and atrophy of the lower limb muscle groups<br>Hands deformity (claw hands)<br>Gait (Steppage)<br>Pyramidal tract damage (bilateral Babinski signs, hypertonia, and hyperreflexia)
Sun et al. 2017[48]	CMT2	NA/AD	1 patient	Missense	Mild muscle atrophy and weakness in lower limbs<br>Foot deformity

					Hyporeflexia at lower limbs
Musacchio et al. 2017[49]	SS	270685/AD	3 patients	Missense	Weakness and atrophy of the distal muscle groups<br>Spasticity<br>Gait (Steppage)<br>Pyramidal tract damage (Brisk tendon reflexes in lower limbs)<br>Sensory impairment
	dHMN-V	619112/AD	2 patients	Missense	Weakness and atrophy of the upper limb muscle groups<br>Pyramidal tract damage (Brisk tendon reflexes)<br>Sensory impairment (Moderate vibration sense impairment)<br>Fasciculations
	SS/ALS	NA/AD	1 patient	Missense	Weakness and atrophy of the distal muscle groups<br>Gait (Steppage, wheelchair)<br>Pyramidal tract damage (Brisk tendon reflexes)<br>Fasciculations<br>Others (dysphagia, respiratory weakness with non-invasive ventilation, and weight loss)
	Subclinical	NA/AD	1 patient	Missense	Fasciculations, cramps, intermittent fasciculation, multiple carcinomas, GQ1b-antibodies
Minami et al. 2018[50]	dHMN with spasticity	NA/AD	1 patient	Missense	Weakness and atrophy of the distal muscle groups<br>Foot deformity (pes cavus)<br>Pyramidal tract damage (Trömner sign and bilateral hyperreflexia)
Fernández-Marmiesse et al. 2019[51]	Severe intractable epilepsy and neurological regression	NA/AD	2 patients	Missense	Epilepsy (febrile and afebrile multiple generalised tonic-clonic seizures, asymmetric tonic seizures, and status epilepticus)<br>Neurodegenerative phenotypes (moderate intellectual disability and autism spectrum disorder)
Mohsenpour et al. 2019[52]	CMT2	NA/AD	3 patients	Missense	Weakness and atrophy of the distal muscle groups<br>Hands deformity (claw hands)<br>Feet deformity (foot drop, hammer toe, and equinovarus)<br>Gait (steppage, unsteady gait and walking frequent falls)<br>Pyramidal tract damage (Babinski signs, hyperreflexia)<br>Others (delayed wound healing, scoliosis, and reduced gag reflex)
Ishihara et al. 2020[53]	CMT	NA/AD	2 patients	Missense	Weakness and atrophy of the distal muscle groups (upper limbs predominate involved)<br>Abnormal knee and ankle reflexs<br>Sensory loss<br>Others (vocal cord paresis, respiratory dysfunction and demyelinating neuropathy)

Ramos-Lopes et al. 2021[54]	SS	270685/AD	1 patient	Missense	Weakness and atrophy of the distal muscle groups<br>Feet deformity with pain (pes cavus with equinus deformity, bilateral claw toes)<br>Gait (scissoring, spastic)<br>Pyramidal tract damage (lower limb hyperreflexia)<br>Others (urinary incontinence)
Stanley et al. 2022[55]	Early-Onset Epileptic Encephalopathy	NA/AD	1 patient	NA	Early-onset epileptic

Abbreviations: CGL2: type 2 congenital generalized lipodystrophy, AR: autosomal recessive; NA: not available, AD: autosomal dominant, dHMN-V: distal hereditary motor neuropathy type V, SS: Silver spastic paraplegia syndrome, CMT: Charcot–Marie–Tooth disease, ALS: Amyotrophic lateral sclerosis, PELD: Celia's encephalopathy or progressive encephalopathy with or without lipodystrophy.

References

1. Rajab, A.; Heathcote, K.; Joshi, S.; Jeffery, S.; Patton, M. Heterogeneity for congenital generalized lipodystrophy in seventeen patients from Oman. *Am. J. Med Genet.* **2002**, *110*, 219–225, https://doi.org/10.1002/ajmg.10437.
2. Bhayana, S.; Siu, V.M.; I Joubert, G.; Clarson, C.L.; Cao, H.; A Hegele, R. Cardiomyopathy in congenital complete lipodystrophy. *Clin. Genet.* **2002**, *61*, 283–287, https://doi.org/10.1034/j.1399-0004.2002.610407.x.
3. Ebihara, K.; Kusakabe, T.; Masuzaki, H.; Kobayashi, N.; Tanaka, T.; Chusho, H.; Miyanaga, F.; Miyazawa, T.; Hayashi, T.; Hosoda, K.; et al. Gene and Phenotype Analysis of Congenital Generalized Lipodystrophy in Japanese: A Novel Homozygous Nonsense Mutation in Seipin Gene. *J. Clin. Endocrinol. Metab.* **2004**, *89*, 2360–2364, https://doi.org/10.1210/jc.2003-031211.
4. Raygada, M.; Rennert, O. Congenital generalized lipodystrophy: profile of the disease and gender differences in two siblings. *Clin. Genet.* **2004**, *67*, 98–101, https://doi.org/10.1111/j.1399-0004.2004.00372.x.
5. Mandal, K.; Aneja, S.; Seth, A.; Khan, A. Berardinelli-Seip congenital lipodystrophy.. *Indian Pediatr.* **2006**, *43*, 440–5.
6. Jin, J.; Cao, L.; Zhao, Z.; Shen, S.; Kiess, W.; Zhi, D.; Ye, R.; Cheng, R.; Chen, L.; Yang, Y.; et al. Novel BSCL2 gene mutation E189X in Chinese congenital generalized lipodystrophy child with early onset diabetes mellitus. *Eur. J. Endocrinol.* **2007**, *157*, 783–787, https://doi.org/10.1530/eje-07-0393.
7. Shirwalkar, H.U.; Patel, Z.M.; Magré, J.; Hilbert, P.; Van Maldergem, L.; Mukhopadhyay, R.R.; Maitra, A. Congenital generalized lipodystrophy in an Indian patient with a novel mutation in BSCL2 gene. *J. Inherit. Metab. Dis.* **2008**, *31*, 317–322, https://doi.org/10.1007/s10545-008-0899-5.
8. Friguls, B.; Coroleu, W.; del Alcazar, R.; Hilbert, P.; Van Maldergem, L.; Pintos-Morell, G. Severe cardiac phenotype of Berardinelli-Seip congenital lipodystrophy in an infant

with homozygous E189X BSCL2 mutation. *Eur. J. Med Genet.* **2009**, *52*, 14–16, https://doi.org/10.1016/j.ejmg.2008.10.006.
9. Miranda, D.M.; Wajchenberg, B.L.; Calsolari, M.R.; Aguiar, M.J.; Silva, J.M.C.L.; Ribeiro, M.G.; Fonseca, C.; Amaral, D.; Boson, W.L.; Resende, B.A.; et al. Novel mutations of the BSCL2 and AGPAT2 genes in 10 families with Berardinelli-Seip congenital generalized lipodystrophy syndrome. *Clin. Endocrinol.* **2009**, *71*, 512–517, https://doi.org/10.1111/j.1365-2265.2009.03532.x.
10. Wu, Y.-R.; Hung, S.-I.; Chang, Y.-C.; Chen, S.-T.; Lin, Y.-L.; Chung, W.-H. Complementary mutations in seipin gene in a patient with Berardinelli-Seip congenital lipodystrophy and dystonia: phenotype variability suggests multiple roles of seipin gene. *J. Neurol. Neurosurg. Psychiatry* **2009**, *80*, 1180–1181, https://doi.org/10.1136/jnnp.2008.165977.
11. Roth, T.; Nair, S.; Kumar, A. Monogenic Diabetes Secondary to Congenital Lipodystrophy in a 14-year-old Yemeni Girl-Case Report. **2010**, *2*, 176–179, https://doi.org/10.4274/jcrpe.v2i4.176.
12. Huang, H.-H.; Chen, T.-H.; Hsiao, H.-P.; Huang, C.-T.; Wang, C.-C.; Shiau, Y.-H.; Chao, M.-C. A Taiwanese Boy With Congenital Generalized Lipodystrophy Caused by Homozygous Ile262fs Mutation in the BSCL2 Gene. *Kaohsiung J. Med Sci.* **2010**, *26*, 615–620, https://doi.org/10.1016/s1607-551x(10)70094-2.
13. Jeninga, E.H.; De Vroede, M.; Hamers, N.; Breur, J.M.P.J.; Verhoeven-Duif, N.M.; Berger, R.; Kalkhoven, E. A Patient with Congenital Generalized Lipodystrophy Due To a Novel Mutation in BSCL2: Indications for Secondary Mitochondrial Dysfunction. **2011**, *4*, 47–54, https://doi.org/10.1007/8904_2011_86.
14. Rahman, O.U.; Khawar, N.; Khan, M.A.; Ahmed, J.; Khattak, K.; Al-Aama, J.Y.; Naeem, M.; Jelani, M. Deletion mutation in BSCL2 gene underlies congenital generalized lipodystrophy in a Pakistani family. *Diagn. Pathol.* **2013**, *8*, 78–78, https://doi.org/10.1186/1746-1596-8-78.
15. Jiang, M.; Gao, M.; Wu, C.; He, H.; Guo, X.; Zhou, Z.; Yang, H.; Xiao, X.; Liu, G.; Sha, J. Lack of testicular seipin causes teratozoospermia syndrome in men. *Proc. Natl. Acad. Sci.* **2014**, *111*, 7054–7059, https://doi.org/10.1073/pnas.1324025111.
16. Schuster, J.; Khan, T.N.; Tariq, M.; Shaiq, P.A.; Mäbert, K.; Baig, S.M.; Klar, J. Exome sequencing circumvents missing clinical data and identifies a BSCL2 mutation in congenital lipodystrophy. *BMC Med Genet.* **2014**, *15*, 71–71, https://doi.org/10.1186/1471-2350-15-71.
17. Haghighi, A.; Kavehmanesh, Z.; Salehzadeh, F.; Santos-Simarro, F.; Van Maldergem, L.; Cimbalistiene, L.; Collins, F.; Chopra, M.; Al Sinani, S.; Dastmalchian, S.; et al. Congenital generalized lipodystrophy: identification of novel variants and expansion of clinical spectrum. *Clin. Genet.* **2015**, *89*, 434–441, https://doi.org/10.1111/cge.12623.
18. Bhujel, N.; Clark, H. Case report: Dental management of Berardinelli-Seip congenital lipodystrophy. *Eur. Arch. Paediatr. Dent.* **2015**, *17*, 137–140, https://doi.org/10.1007/s40368-015-0210-z.

19. Opri, R.; Fabrizi, G.M.; Cantalupo, G.; Ferrarini, M.; Simonati, A.; Bernardina, B.D.; Darra, F. Progressive Myoclonus Epilepsy in Congenital Generalized Lipodystrophy type 2: Report of 3 cases and literature review. *Seizure* **2016**, *42*, 1–6, https://doi.org/10.1016/j.seizure.2016.08.008.
20. Teboul-Coré, S.; Rey-Jouvin, C.; Miquel, A.; Vatier, C.; Capeau, J.; Robert, J.-J.; Pham, T.; Lascols, O.; Berenbaum, F.; Laredo, J.-D.; et al. Bone imaging findings in genetic and acquired lipodystrophic syndromes: an imaging study of 24 cases. *Skelet. Radiol.* **2016**, *45*, 1495–1506, https://doi.org/10.1007/s00256-016-2457-9.
21. Su, X.; Lin, R.; Huang, Y.; Sheng, H.; Li, X.; Ting, T.H.; Liu, L.; Li, X. Clinical and Mutational Features of Three Chinese Children with Congenital Generalized Lipodystrophy. *J. Clin. Res. Pediatr. Endocrinol.* **2017**, *9*, 52–57, https://doi.org/10.4274/jcrpe.3556.
22. Chen, R.; Yuan, X.; Wang, J.; Zhang, Y. Clinical and molecular characterization of two Chinese patients with Type 2 congenital generalized lipodystrophy. *Gene* **2017**, *637*, 57–62, https://doi.org/10.1016/j.gene.2017.09.023.
23. Lima, J.G.; Nobrega, L.H.C.; Lima, N.N.; dos Santos, M.C.F.; Baracho, M.D.F.P.; Winzenrieth, R.; Bandeira, F.; Mendes-Aguiar, C.D.O.; Neto, F.P.F.; Ferreira, L.C.; et al. Normal bone density and trabecular bone score, but high serum sclerostin in congenital generalized lipodystrophy. *Bone* **2017**, *101*, 21–25, https://doi.org/10.1016/j.bone.2017.03.053.
24. Akinci, G.; Topaloglu, H.; Demir, T.; Danyeli, A.E.; Talim, B.; Keskin, F.E.; Kadioglu, P.; Talip, E.; Altay, C.; Yaylali, G.F.; et al. Clinical spectra of neuromuscular manifestations in patients with lipodystrophy: A multicenter study. *Neuromuscul. Disord.* **2017**, *27*, 923–930, https://doi.org/10.1016/j.nmd.2017.05.015.
25. Purizaca-Rosillo, N.; Mori, T.; Benites-Cóndor, Y.; Hisama, F.M.; Martin, G.M.; Oshima, J. High incidence ofBSCL2intragenic recombinational mutation in Peruvian type 2 Berardinelli-Seip syndrome. *Am. J. Med Genet. Part A* **2016**, *173*, 471–478, https://doi.org/10.1002/ajmg.a.38053.
26. Gonzalo, M.M.; Estefania, C.V. Congenital Generalized Lipodystrophy Type 2 in a Patient From a High-Prevalence Area.. *J. Endocr. Soc.* **2017**, *1*, 1012–1014, https://doi.org/10.1210/js.2017-00141.
27. Ponte, C.M.M.; Fernandes, V.O.; Gurgel, M.H.C.; Vasconcelos, I.T.G.F.; Karbage, L.B.D.A.S.; Liberato, C.B.R.; Negrato, C.A.; Gomes, M.D.B.; Montenegro, A.P.D.R.; Júnior, R.M.M. Early commitment of cardiovascular autonomic modulation in Brazilian patients with congenital generalized lipodystrophy. *BMC Cardiovasc. Disord.* **2018**, *18*, 6, https://doi.org/10.1186/s12872-017-0738-4.
28. Liu, Y.; Li, D.; Ding, Y.; Kang, L.; Jin, Y.; Song, J.; Li, H.; Yang, Y. Further delineation of AGPAT2 and BSCL2 related congenital generalized lipodystrophy in young infants. *Eur. J. Med Genet.* **2018**, *62*, 103542, https://doi.org/10.1016/j.ejmg.2018.09.009.
29. Lima, G.E.D.C.P.; Fernandes, V.O.; Montenegro, A.P.D.R.; De Carvalho, A.B.; Karbage, L.B.D.A.S.; Aguiar, L.B.; Macedo, M.S.R.; Ferreira, L.A.A.; Júnior, R.M.M.; Montenegro, R.M. Aggressive papillary thyroid carcinoma in a child with type 2 congenital

generalized lipodystrophy. *Arch. Endocrinol. Metab.* **2019**, *63*, 79–83, https://doi.org/10.20945/2359-3997000000096.
30. Zhang, Y.; Chen, X.; Luo, F.; Jiang, L.; Xu, J.; Chen, S. Medical management of a child with congenital generalized lipodystrophy accompanied with progressive myoclonic epilepsy. *Medicine* **2019**, *98*, e18121, https://doi.org/10.1097/md.0000000000018121.
31. Serino, D.; Davico, C.; Specchio, N.; Marras, C.E.; Fioretto, F. Berardinelli-Seip syndrome and progressive myoclonus epilepsy. Epileptic disorders : international epilepsy journal with videotape 2019, 21, 117-121, doi:10.1684/epd.2019.1038.
32. Qin, Y.-Y.; Zhang, X.; Xiang, L.-Q.; Shan, Q.-W.; Li, S.-D.; Yan, J.; Lin, F.-Q. A New Compound Heterozygous Mutation Of *BSCL2* In A Chinese Zhuang Ethnic Family With Congenital Generalized Lipodystrophy. *Diabetes, Metab. Syndr. Obesity: Targets Ther.* **2019**, *ume 12*, 2583–2587, https://doi.org/10.2147/dmso.s207293.
33. Yamamoto, A.; Kusakabe, T.; Sato, K.; Tokizaki, T.; Sakurai, K.; Abe, S. Seipin-linked congenital generalized lipodystrophy type 2: a rare case with multiple lytic and pseudo-osteopoikilosis lesions. *Acta Radiol. Open* **2019**, *8*, https://doi.org/10.1177/2058460119892407.
34. Sánchez-Iglesias, S.; Crocker, M.; O'Callaghan, M.; Darling, A.; García-Cazorla, A.; Domingo-Jiménez, R.; Castro, A.; Fernández-Pombo, A.; Ruibal, .; Aguiar, P.; et al. Celia's encephalopathy and c.974dupG in BSCL2 gene: a hidden change in a known variant. *neurogenetics* **2019**, *20*, 73–82, https://doi.org/10.1007/s10048-019-00574-5.
35. Poisson, A.; Chatron, N.; Labalme, A.; Till, M.; Broussolle, E.; Sanlaville, D.; Demily, C.; Lesca, G. Regressive Autism Spectrum Disorder Expands the Phenotype of BSCL2/Seipin-Associated Neurodegeneration. *Biol. Psychiatry* **2018**, *85*, e17–e19, https://doi.org/10.1016/j.biopsych.2018.05.010.
36. Ferranti, S.; Rizzo, C.L.; Renieri, A.; Galluzzi, P.; Grosso, S. Focus on progressive myoclonic epilepsy in Berardinelli-Seip syndrome. *Neurol. Sci.* **2020**, *41*, 3345–3348, https://doi.org/10.1007/s10072-020-04418-1.
37. Yang, Y.; Ma, L.; Sun, J.; Gong, X.; Cai, C.; Hong, W. The neonatal onset diabetes mellitus of Chinese neonate with congenital generalized lipodystrophy 2: a case report. *BMC Endocr. Disord.* **2022**, *22*, 1–5, https://doi.org/10.1186/s12902-022-00992-x.
38. Rohkamm, B.; Reilly, M.M.; Lochmüller, H.; Schlotter-Weigel, B.; Barisic, N.; Schöls, L.; Nicholson, G.; Pareyson, D.; Laurà, M.; Janecke, A.R.; et al. Further evidence for genetic heterogeneity of distal HMN type V, CMT2 with predominant hand involvement and Silver syndrome. *J. Neurol. Sci.* **2007**, *263*, 100–106, https://doi.org/10.1016/j.jns.2007.06.047.
39. Bienfait, H.; Baas, F.; Koelman, J.H.; de Haan, R.J.; van Engelen, B.G.; Gabreels-Festen, A.A.; de Visser, B.W.O.; Meggouh, F.; Weterman, M.A.; De Jonghe, P.; et al. Phenotype of Charcot-Marie-Tooth disease Type 2. *Neurology* **2007**, *68*, 1658–1667, https://doi.org/10.1212/01.wnl.0000263479.97552.94.
40. Cho, H.-J.; Sung, D.-H.; Ki, C.-S. Identification of de novoBSCL2 Ser90Leu mutation in a Korean family with silver syndrome and distal hereditary motor neuropathy. *Muscle Nerve* **2007**, *36*, 384–386, https://doi.org/10.1002/mus.20792.

41. Cafforio, G.; Calabrese, R.; Morelli, N.; Mancuso, M.; Piazza, S.; Martinuzzi, A.; Bassi, M.T.; Crippa, F.; Siciliano, G. The first Italian family with evidence of pyramidal impairment as phenotypic manifestation of Silver syndrome BSCL2 gene mutation. *Neurol. Sci.* **2008**, *29*, 189–191, https://doi.org/10.1007/s10072-008-0937-y.
42. Chen, B.; Zheng, R.; Luan, X.; Zhang, W.; Wang, Z.; Yuan, Y. Clincial and pathological study of distal motor neuropathy with N88S mutation in BSCL2. *Neuropathology* **2009**, *29*, 543–547, https://doi.org/10.1111/j.1440-1789.2009.01011.x.
43. Rakočević-Stojanović, V.; Milić-Rašić, V.; Perić, S.; Baets, J.; Timmerman, V.; Dierick, I.; Pavlović, S.; De Jonghe, P. N88S mutation in the BSCL2 gene in a Serbian family with distal hereditary motor neuropathy type V or Silver syndrome. *J. Neurol. Sci.* **2010**, *296*, 107–109, https://doi.org/10.1016/j.jns.2010.06.015.
44. Luigetti, M.; Fabrizi, G.; Madia, F.; Ferrarini, M.; Conte, A.; Delgrande, A.; Tonali, P.; Sabatelli, M. SEIPIN S90L Mutation in an Italian family with CMT2/dHMN and pyramidal signs. *Muscle Nerve* **2010**, *42*, 448–451, https://doi.org/10.1002/mus.21734.
45. Cen, Z.; Lu, X.; Wang, Z.; Ouyang, Z.; Xie, F.; Luo, W. BSCL2 S90L mutation in a Chinese family with Silver syndrome with a review of the literature. *J. Clin. Neurosci.* **2014**, *22*, 429–430, https://doi.org/10.1016/j.jocn.2014.08.010.
46. Ollivier, Y.; Magot, A.; Latour, P.; Perrier, J.; Mercier, S.; Maisonobe, T.; Péréon, Y. Clinical and electrophysiological features in a French family presenting with seipinopathy. *Neuromuscul. Disord.* **2014**, *25*, 161–164, https://doi.org/10.1016/j.nmd.2014.10.006.
47. Hsiao, C.-T.; Tsai, P.-C.; Lin, C.-C.; Liu, Y.-T.; Huang, Y.-H.; Liao, Y.-C.; Huang, H.-W.; Lin, K.-P.; Soong, B.-W.; Lee, Y.-C. Clinical and Molecular Characterization of BSCL2 Mutations in a Taiwanese Cohort with Hereditary Neuropathy. *PLoS ONE* **2016**, *11*, e0147677–e0147677, https://doi.org/10.1371/journal.pone.0147677.
48. Sun, B.; Chen, Z.; Ling, L.; Yang, F.; Huang, X. Clinical and genetic spectra of Charcot-Marie-Tooth disease in Chinese Han patients. *J. Peripher. Nerv. Syst.* **2017**, *22*, 13–18, https://doi.org/10.1111/jns.12195.
49. Musacchio, T.; Zaum, A.-K.; Üçeyler, N.; Sommer, C.; Pfeifroth, N.; Reiners, K.; Kunstmann, E.; Volkmann, J.; Rost, S.; Klebe, S. ALS and MMN mimics in patients with BSCL2 mutations: the expanding clinical spectrum of SPG17 hereditary spastic paraplegia. *J. Neurol.* **2016**, *264*, 11–20, https://doi.org/10.1007/s00415-016-8301-2.
50. Minami, K.; Takahashi, S.; Nihei, Y.; Oki, K.; Suzuki, S.; Ito, D.; Takashima, H.; Suzuki, N. The First Report of a Japanese Case of Seipinopathy with a BSCL2 N88S Mutation. **2018**, *57*, 613–615, https://doi.org/10.2169/internalmedicine.8765-16.
51. Fernández-Marmiesse, A.; Sánchez-Iglesias, S.; Darling, A.; O'Callaghan, M.M.; Tonda, R.; Jou, C.; Araújo-Vilar, D. A de novo heterozygous missense BSCL2 variant in 2 siblings with intractable developmental and epileptic encephalopathy. *Seizure* **2019**, *71*, 161–165, https://doi.org/10.1016/j.seizure.2019.07.019.
52. Mohsen-Pour, N.; Rokni-Zadeh, H.; Maghbooli, M.; Changi-Ashtiani, M.; Shahrooei, M.; Salehi, M.; Behnam, M.; Shahani, T.; Biglari, A. Whole Exome Sequencing Revealed a Novel GJB1 Pathogenic Variant and a Rare BSCL2 Mutation in Two Iranian Large

Pedigrees with Multiple Affected Cases of Charcot-Marie-Tooth. **2019**, *8*, 169–178, https://doi.org/10.22088/IJMCM.BUMS.8.3.169.
53. Ishihara, S.; Okamoto, Y.; Tanabe, H.; Yoshimura, A.; Higuchi, Y.; Yuan, J.; Hashiguchi, A.; Ishiura, H.; Mitsui, J.; Suwazono, S.; et al. Clinical features of inherited neuropathy with BSCL2 mutations in Japan. *J. Peripher. Nerv. Syst.* **2020**, *25*, 125–131, https://doi.org/10.1111/jns.12369.
54. Ramos-Lopes, J.; Ribeiro, J.; Laço, M.; Alves, C.; Matos, A.; Costa, C. A De Novo BSCL2 Gene S90L Mutation in a Progressive Tetraparesis with Urinary Dysfunction and Corpus Callosum Involvement. *J. Pediatr. Genet.* **2020**, *10*, 253–258, https://doi.org/10.1055/s-0040-1713768.
55. E Stanley, N.; Robinson, L.J.; Mao, Q. A Novel, Heterozygous *BSCL2* Variant in Association With Early-Onset Epileptic Encephalopathy. *J. Neuropathol. Exp. Neurol.* **2022**, *81*, 377–380, https://doi.org/10.1093/jnen/nlac013.
